# Clinical efficacy of comprehensive rehabilitation intervention and its effect on Quality of Life in patients with Advanced Liver Cancer after Ultrasound-guided Microwave Ablation

**DOI:** 10.12669/pjms.39.3.6663

**Published:** 2023

**Authors:** Dan Chen, Fan Yang, Xiu-ju Wang, Jing Zhao

**Affiliations:** 1 Dan Chen, Interventional Ultrasound Department, Department of Oncology, The Fifth Medical Center of Chinese PLA General Hospital, Beijing 100039, P. R. China; 2 Fan Yang, Department of Medical Oncology, Department of Oncology, The Fifth Medical Center of Chinese PLA General Hospital, Beijing 100039, P. R. China; 3 Xiu-ju Wang, Department of Medical Oncology, Department of Oncology, The Fifth Medical Center of Chinese PLA General Hospital, Beijing 100039, P. R. China; 4 Jing Zhao, Department of Anesthesiology, The First Medical Center of Chinese PLA General Hospital, Beijing 100853, P. R. China

**Keywords:** Rehabilitation, Liver neoplasms, Quality of life, Complications

## Abstract

**Objective:**

To evaluate the clinical efficacy of comprehensive rehabilitation intervention and its effect on the quality of life (QoL) in patients with advanced liver cancer after ultrasound-guided microwave ablation (UMA).

**Methods:**

This is a retrospective study. Total 110 in-patients with advanced liver cancer who had received UMA in our hospital from January 2019 to January 2021 were included and randomly divided into two groups. Patients in the control group received the conventional intervention and those in the experimental group received comprehensive rehabilitation intervention. The incidence of postoperative complications as well as the differences in indicators, including emotional status, QoL score, and patient satisfaction before and after the intervention were analyzed and compared between the two groups. The differences in survival between the two groups were compared.

**Results:**

The incidence of postoperative complications in the experimental group was significantly lower than that of the control group. SAS and SDS scores of the experimental group were significantly decreased after intervention, while the control group had no significant change before and after intervention. KPS and SF-36 quality of life scores in the experimental group were significantly improved compared with the control group, and patient satisfaction was significantly higher than the control group, and the 12-month survival rate was significantly higher than that in the control group.

**Conclusion:**

Comprehensive rehabilitation intervention can reduce the incidence of postoperative complications, improve the mood and QoL, and increase patient satisfaction and survival rate in patients with advanced liver cancer after UMA.

## INTRODUCTION

Primary liver cancer is a common malignancy with the third-highest mortality that is only second to lung cancer and gastric cancer.^1^ There are no specific symptoms in the early stage of this disease, and most patients are in the advanced stages when diagnosed with a poor prognosis.^2^ According to the International Cancer Research Center of the World Health Organization, the current death/new cases of liver cancer patients are as high as 97%.^3^ For liver cancer patients who are not eligible for surgical treatment, interventional treatment methods such as ultrasound-guided microwave ablation (UMA) are mainly used currently. Clinical studies have found that UMA can effectively improve the survival and signs and prolong the survival of advanced patients.^4^ However, microwave ablation is associated with significant adverse reactions and post procedural complications, which impairs the prognosis of patients.^5^ At the same time, patients often suffer a serious psychological burden and are prone to negative emotions such as anxiety, depression, fear, and tension due to the missed opportunity for surgery. These emotions will further aggravate the patient’s condition. This mutual influence impairs the QoL of the patients and causes huge losses to the patient’s family and even society.^6^ Comprehensive rehabilitation intervention refers to medical means and measures to comprehensively deal with risk factors such as adverse behaviors, unhealthy lifestyles and habits that affect health and the resulting poor health status, and includes health counseling and health education, nutrition and exercise intervention, psychological and mental health intervention, health risk control and management, and medical guidance among others.^7^ Evidence supports that this approach can effectively relieve anxiety and depression, with the potential to restore physical and mental states to precancerous levels.^8^

Currently, there are no reports on the effect of “comprehensive rehabilitation intervention” on postoperative complications and QoL in patients with advanced liver cancer after microwave ablation. In this study, comprehensive rehabilitation intervention was applied in these patients and clinical therapeutic effect was obtained.

## METHODS

This is a retrospective study. A total of 110 patients with advanced liver cancer who underwent UMA in our hospital from January 2019 to January 2021 were selected and randomly divided into two groups, with 55 patients in each group. There were 35 males and 20 females in the experimental group, with an average age of 65.48±8.05 years old (range 57-73 years), and 33 males and 22 females in the control group, with an average of 64.57±8.33 years old (range 54-75 years). The two groups were comparable with no significant differences in general data (Table-I). *The* study was approved by the Institutional Ethics Committee of The First Medical Center of Chinese PLA General Hospital on April 10, 2019 (No. [2019]043), and written informed consent was obtained from all participants.

**Table-I:** Comparative Analysis of general data between the experimental group and the control group(*χ̅*±*S*) (n=55 for each group).

Items	Experimental group	Control group	t/χ^2^	P
Age (years)	65.48±8.05	64.57±8.33	0.58	0.56
Male (n, %)	35(63.6%)	33(60%)	0.15	0.70
Advanced (n, %)	21(38.2%)	25(45.5%)	0.60	0.44
Course of disease (Months)	7.25±2.33	7.09±2.16	0.37	0.71
Tumor diameter (mm)	51.24±11.85	50.76±10.27	0.23	0.82

P>0.05

### Inclusion criteria:

- Met the diagnostic criteria for advanced liver cancer^9^,
- <75 years old,
- With the ability of self-care (KPS score ≥80)^10^,
- With complete clinical data,
- Able to cooperate to complete the study with good compliance.

### Exclusion criteria:

- Malignant tumors in other sites of the body at the same time,
- Severe organ dysfunction,
- Coagulation and endocrine diseases,
- Metastatic liver cancer,
- Cognitive or behavioral abnormalities that prevented the completion of the study.

### Treatments:

Patients in the control group were given conventional intervention during the perioperative period, including (1) preoperative health education, psychological intervention, and specific guidance and explanation; and postoperative close observation, symptomatic treatment and consolation; (2) intraoperative intervention, including psychological counseling, observation of the condition, and symptomatic treatment; and (3) Observation of the postoperative symptoms such as fever, pain, constipation, abdominal distension, nausea and vomiting as well as symptomatic treatment given when symptoms occurred.

Patients in the experimental group were given a comprehensive rehabilitation intervention in addition to the intervention the control group received, and mainly included evaluation of the patient’s general health, conditions and psychological state by the attending doctor to develop rehabilitation measures; psychological rehabilitation intervention which were jointly implemented by the attending doctor and the nurse in charge; and early postoperative exercise and rehabilitation training to promote recovery. ***Health education:*** medical professionals explained the UMA for liver cancer, relevant precautions and possible complications to the patients in detail, and purposefully carried out defecation training in bed before the operation to enhance the confidence of the patients. ***Psychological rehabilitation measures***: the patient’s emotional changes were carefully observed to relieve negative emotions, and trust was created through good communication to relieve anxiety and fear, and therefore to improve treatment compliance. Patients were instructed to build the confidence to overcome the disease and improve their psychological resistance. ***Nutritional intervention:*** The adverse reactions were serious after microwave ablation and symptomatic treatment was provided timely with light and nutritious food. Irritating food was prohibited and drinking more water was encouraged. Oral care was provided to remove oral odor, which was beneficial for the patient’s condition. ***Exercise instruction:*** Appropriate physical exercise based on the characteristics was instructed to enhance physical fitness and build up resistance to diseases. ***Prognosis observation:*** vital signs and therapeutic effects were closely observed for the patients to prevent the development of various complications and actively deal with them.

### Outcome Measures:

***The incidence of postoperative complications:*** The perioperative complications such as abdominal pain, nausea and vomiting, gastrointestinal bleeding, and constipation were recorded for the two groups of patients, and the incidence of postoperative complications was compared between the two groups. ***Analysis of emotional status:*** the self-rating anxiety scale (SAS) and self-rating depression scale (SDS)^11^ were used to evaluate the emotional changes of the two groups before and after the intervention. A lower score indicated better emotional status. (3) QoL scores: Karnofsky performance score (KPS scale) and quality of life (SF-36 QoL scale) score were used to compare and analyze the improvement of QoL of the two groups of patients before and after the intervention. ***Comparative analysis of patient satisfaction:*** the patient satisfaction questionnaire short form (PSQ-18)^12^ was used to comparatively analyze the patient satisfaction before and after the intervention. Scores included very satisfied, somewhat satisfied, satisfied, uncertain, and dissatisfied, and the overall satisfaction rate was calculated as (very satisfied + somewhat satisfied + satisfied)/total number of patients x 100%. ***Comparative analysis of follow-up results:*** *T*he two groups of patients were followed up for 12 months, and the differences in survival rates between the two groups were analyzed and compared.

### Statistical Analysis:

All data were analyzed by SPSS 20.0 software. Measurement data were presented as (*χ̅*±*s*). Data were analyzed by two independent-sample t-tests between the groups and by paired t-test within the groups. Rates were compared by χ^2^ tests. P<0.05 was considered statistically significant.

## RESULTS

The incidence of postoperative complications in the experimental group was 20%, which was significantly lower than that of the control group (43%), and the difference was statistically significant (p=0.04) (Table-II).

**Table-II:** Comparative analysis of the incidence of postoperative complications between the two groups (*χ̅*±*S*) (n=55 for each group).

Groups	Abdominal pain	Nausea/Vomiting	Abdominal distention	Constipation	Incidence
Experimental. group	4	3	4	0	11(20%)
Control group	5	6	9	3	23(42%)
c^2^					4.17
P					0.04

P<0.05.

Both SAS and SDS in the experimental group were significantly decreased after the intervention compared with those before the intervention with statistically significant differences (p=0.00). No significant changes in SAS and SDS scores were observed in the control group before and after the intervention (P>0.05) (Table-III).

**Table-III:** Comparative analysis of the emotional state of the two groups before and after the intervention (*χ̅*±*S*) (n=55 for each group).

Measurements		Experimental Group*	Control group	t	p
SAS	Before intervention	63.27±7.83	62.79±7.45	0.33	0.74
	After intervention*	42.46±6.23	61.82±6.73	15.66	0.00
	t	15.42	0.74		
	p	0.00	0.46		
SDS	Before intervention	66.73±7.38	66.52±7.59	0.15	0.88
	After intervention *	47.06±7.33	65.79±7.85	12.93	0.00
	t	14.02	0.50		
	p	0.00	0.62		

***Notes:***

* p<0.05.

There were no significant differences in KPS score and SF-36 QoL score between the two groups before the intervention (p>0.05). After the intervention, both scores were significantly improved in the experimental group compared with those in the control group, and the differences were statistically significant (p=0.00) (Table-IV).

**Table-IV:** Comparative analysis of the QoL scores in the two groups before and after the intervention (*χ̅*±*S*) (n=55 for each group).

Measurements		Experimental group	Control group	t	p
KPS Score	Before intervention	67.32±11.28	67.29±12.10	0.01	0.98
	After intervention*	92.71±12.07	83.25±12.63	4.02	0.00
SF-36 Score	Before intervention	46.58±5.27	47.02±5.13	0.44	0.65
	After intervention*	80.40±7.84	71.35±7.41	6.22	0.00

***Notes:***

* p<0.05.

**Fig.1:**
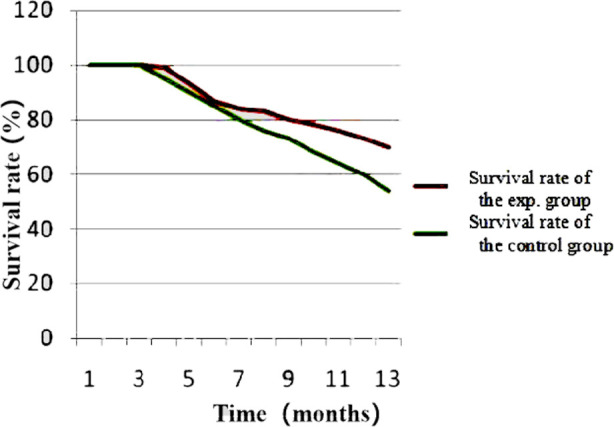
Comparative analysis of survival rate between the two groups.

Patient satisfaction rate in the experimental group was 96.4%, which was higher than that in the control group (83.6%), and the difference was statistically significant (p=0.02) (Table-V). The follow-up results showed that the survival rate was 73% in the experimental group and 56.4% in the control group, and the difference was statistically significant (c^2^=3.93, p=0.03) (Fig.1).

**Table-V:** Comparative analysis of patient satisfaction between the two groups after the intervention (*χ̅*±*S*±S) (n=55 for each group).

Groups	Very satisfied	Somewhat satisfied	Satisfied	Uncertain	Dissatisfied	Overall satisfaction rate*
Experimental group	36	8	9	2	0	53(96.4%)
Control group	27	11	8	6	3	46(83.6%)
c^2^						4.95
P						0.02

***Notes:***

* p<0.05.

## DISCUSSION

Liver cancer is a common malignancy of the digestive system with a high mortality rate.^13^ There are no specific clinical manifestations in the early stage, and symptoms such as liver pain, anorexia, and abdominal distension are usually associated with the advanced stages of the disease.^14^ Less than 20% of patients are eligible for surgical treatment, and more than 80% of patients have missed the opportunity for operation. For patients with inoperable liver cancer, UMA is one of the main treatment options.^15^ Negative and despair thoughts such as tension, anxiety, depression, and fear may occur due to the fact that patients are not familiar with the disease and the microwave ablation scheme and worry about the prognosis of the disease and the chemotherapy-induced adverse reactions. Patients suffer from significant mental stress, which may seriously impair the treatment effect, harm the recovery of the disease, and reduce the compliance with UMA treatment, thereby affecting the QoL of the patients. Therefore, the improvement of mental health and QoL in the treatment of malignant tumors in clinical practice is of great importance for prognosis.^16^

When using the traditional rehabilitation intervention, the medical professionals cannot adjust the intervention measures in a timely manner based on the condition of patient recovery due to the lack of comprehensive understanding of the patient’s psychological needs, disease changes and treatment effects, and fail to continuously control and manage the quality of medical service, which in turn increases the difficulty of rehabilitation training.^17^ Comprehensive rehabilitation intervention is increasingly recognized as an effective tool in dealing with injuries and dysfunctions after cancer treatment.^18^ It can improve the functional capacities of the patients, including coping and adapting to the loss of function, enhancing confidence to challenges and avoiding psychological dependence on others.^19^ Comprehensive rehabilitation intervention is composed of various intervention measures, including health counseling and health education, nutrition and exercise intervention, psychological and mental intervention, health risk control and management, and medical instruction.^20^ Comprehensive rehabilitation intervention is a supplement and continuation of clinical treatments.^21^ Effective comprehensive rehabilitation intervention can reduce morbidity and save medical costs^22^, as well as help patients achieve their personal goals and improve the satisfaction of their life.^23^

**Table:** How can we explain “Prognosis observation” as a part of the comprehensive rehabilitation intervention? (Extra sheet attached for reference).

Conventional Intervention - (Control Arm)	Additional Intervention – Rehabilitation (Experimental arm)
(1) preoperative health education, psychological intervention, and specific guidance and explanation; and postoperative close observation, symptomatic treatment and consolation.	(1) evaluation of the patient’s general health, conditions and psychological state by the attending doctor to develop rehabilitation measures; psychological rehabilitation intervention which were jointly implemented by the attending doctor and the nurse in charge; and early postoperative exercise and rehabilitation training to promote recovery.
(2) intraoperative intervention, including psychological counseling, observation of the condition, and symptomatic treatment; and	(2) Health education: medical professionals explained the UMA for liver cancer, relevant precautions and possible complications to the patients in detail, and purposefully carried out defecation training in bed before the operation to enhance the confidence of the patients.
(3) Observation of the postoperative symptoms such as fever, pain, constipation, abdominal distension, nausea and vomiting as well as symptomatic treatment given when symptoms occurred.	(3) Psychological rehabilitation measures: the patient’s emotional changes were carefully observed to relieve negative emotions, and trust was created through good communication to relieve anxiety and fear, and therefore to improve treatment compliance. Patients were instructed to build the confidence to overcome the disease and improve their psychological resistance.
	(4) Nutritional intervention: The adverse reactions were serious after microwave ablation and symptomatic treatment was provided timely with light and nutritious food. Irritating food was prohibited and drinking more water was encouraged. Oral care was provided to remove oral odor, which was beneficial for the patient’s condition.
	(5) Exercise instruction: Appropriate physical exercise based on the characteristics was instructed to enhance physical fitness and build up resistance to diseases.
	(6) Prognosis observation: vital signs and therapeutic effects were closely observed for the patients to prevent the development of various complications and actively deal with them.

Our results confirmed that comprehensive rehabilitation intervention can reduce the incidence of postoperative complications, improve the mood and QoL, and increase patient satisfaction and survival rate in patients with advanced liver cancer after UMA. Our study can provide some reference for the implementation of comprehensive rehabilitation intervention in patients with advanced liver cancer after microwave ablation. Wang et al^24^ pointed out that comprehensive education and care relieves anxiety and depression, improves quality of life, and prolongs survival in patients with hepatocellular carcinoma underwent surgical resection. EJ et al^25^ found that non-pharmacological interventions using a comprehensive functional rehabilitation programme improve functionality and relieve dyspnoea in cancer patients. According to Hall et al^26^, an exercise and nutritional rehabilitation intervention is feasible and has potential benefits for people with incurable cancer. Research by Egan et al^17^ shows that the efficacy of comprehensive rehabilitation intervention involves many dimensions, including physical function, fatigue, pain, sexual function, cognitive function, depression, employment, and nutrition, and many of them can be improved via rehabilitation intervention, such as pain, sexual function, cognitive function and return to work. Ng et al^27^ believed that comprehensive rehabilitation intervention is beneficial to treatment in many ways. For example, it can increase functional independence and overall emotional and spiritual improvement.

### Limitations of the study:

The sample size was small and the study only involved patients after microwave ablation, which was relatively inadequate. In future clinical studies, the sample size will be increased with extended follow-up time, and the clinical effects of comprehensive rehabilitation intervention combined with other treatment options such as surgery, radiotherapy, chemotherapy or immunotherapy will be included to enrich the study and evaluate the advantages and disadvantages of the intervention program more objectively, so as to benefit more patients.

## CONCLUSION

In summary, comprehensive rehabilitation intervention can safely and effectively decrease the incidence of complications, reduce the stress response, improve mood and QoL, and increase patient satisfaction and survival rate in patients with liver cancer who underwent microwave ablation.

***Authors’ Contributions:***

**JZ:** Designed this study and prepared this manuscript, and are responsible and accountable for the accuracy or integrity of the work.

**DC:** Collected and analyzed clinical data.
